# Laser-Based Ablation of Titanium–Graphite Composite for Dental Application

**DOI:** 10.3390/ma13102312

**Published:** 2020-05-18

**Authors:** Peter Šugár, Barbora Ludrovcová, Jaroslav Kováčik, Martin Sahul, Jana Šugárová

**Affiliations:** 1 Institute of Production Technologies, Faculty of Materials Science and Technology, Slovak University of Technology, J. Bottu 25, 917 24 Trnava, Slovakia; barbora.ludrovcova@stuba.sk (B.L.); jana.sugarova@stuba.sk (J.Š.); 2 Institute of Materials and Machine Mechanics, Slovak Academy of Sciences, Dúbravská cesta 9, 845 13 Bratislava, Slovakia; Jaroslav.Kovacik@savba.sk; 3 Institute of Material Science, Faculty of Materials Science and Technology, Slovak University of Technology, J. Bottu 25, 917 24 Trnava, Slovakia; martin.sahul@stuba.sk

**Keywords:** laser, machining, titanium, composite, powder metallurgy, surface, morphology, implant

## Abstract

Biocompatible materials with excellent mechanical properties as well as sophisticated surface morphology and chemistry are required to satisfy the requirements of modern dental implantology. In the study described in this article, an industrial-grade fibre nanosecond laser working at 1064 nm wavelength was used to micromachine a new type of a biocompatible material, Ti-graphite composite prepared by vacuum low-temperature extrusion of hydrogenated-dehydrogenated (HDH) titanium powder mixed with graphite flakes. The effect of the total laser energy delivered to the material per area on the machined surface morphology, roughness, surface element composition and phases transformations was investigated and evaluated by means of scanning electron microscopy (SEM), energy-dispersive X-ray spectroscopy (EDS), confocal laser-scanning microscopy (CLSM) and X-ray diffraction analysis (XRD). The findings illustrate that the amount of thermal energy put to the working material has a remarkable effect on the machined surface properties, which is discussed from the aspect of the contact properties of dental implants.

## 1. Introduction

Low density, light weight [1,2], corrosion and chemical resistance [3,4], good mechanical properties [5,6,7] and biocompatibility [8,9] are the reasons why titanium (Ti)-based materials are utilized in a wide range of industries such as shipbuilding [10], aerospace [11], aircraft [12] and automotive [13]. It is due to the non-toxicity and biocompatibility that the titanium-based materials are also very popular in dental and orthopaedic implantology [14,15,16,17,18,19]. They are used as a commercially pure (CP) Ti with different degrees of purity influencing their mechanical properties, as multicomponent metallic alloys or as Ti metal matrix composites (TiMMCs) reinforced with different types of particles or whiskers. TiMMCs represent a novel generation of biomaterials with superior properties and cost-effective methods of fabrication compared with conventional metallurgical methods. The strong effort to develop this kind of material by different manufacturing processes with the goal of optimising their structure, mechanical properties and surface modification, is visible in many studies, as documented in the literature review.

Lin et al. [20] produced a Ti–Mg composite with lower compression modulus of 36–50 GPa and high compressive strength of 1500–1800 MPa by combining mechanical alloying and spark plasma sintering. Jiang et al. [21] utilized powder metallurgy (PM) and ultrasonic infiltration for fabrication of the Ti–Mg composites. They documented increasing modulus and strength with decreasing Ti particle size. Balog et al. [22] fabricated a Ti–Mg composite with 12 vol.% Mg by warm extrusion. The developed composite showed density of 4.12 g·cm^−3^, ultimate tensile strength of 529 MPa, while the Young’s elastic modulus was reduced to 92.1 GPa. In their later study [23], they investigated PM TiMMC with 12, 17 and 24 vol.% of Mg, produced by a low-temperature process, where they achieved Young’s modulus of 81 GPa and ultimate tensile strength of 409 MPa. The authors in [24,25,26] have focused on the Ti–CaP composites and studied the influence of the CaP on strength, hardness, wear resistance and bioactivity. The wear rate decreasing by 92% was observed when 10 wt.% of CaP was added. Han et al. [27] developed Ti–hydroxyapatite (HA) with outstanding biocompatibility and bioactivity, prepared by selective laser melting. Ultimate tensile strength of 289 MPa and microhardness of 600.8 HV were reached by adding 2% of nanoscale HA. Miranda et al. [28] also used HA as reinforcement of the Ti6Al4V matrix; however the composite was prepared by hot pressing. The PM methods such as cold pressing and vacuum sintering were used in [29] for production of a Ti–Mn composite with 1.5 and 10 wt.% of Mn, resulting in mechanical properties similar to other biomedical Ti alloys. The Ti–ZrO_2_ composite was developed in [30] by a pressureless sintering process with polymethyl methacrylate (PMMA) as a pore forming agent; the latter was subsequently removed by heat treatment. During the process, connected pores were formed, and the elastic modulus of 22.4 GPa, which is near the cortical bone with elastic modulus of 7–25 GPa, was achieved. The Ti–Nb_2_O_5_ composite was fabricated by the PM process in [31]. With 2, 3 and 4 wt.% of Nb_2_O_5,_ the yield strength of the composite increased to 1245, 1310 and 1409 MPa, respectively. The composites produced exhibited excellent biocompatibility and cell adhesion. In addition to mechanical properties, the surface integrity is also a very important quality factor of biomedical materials. Emphasized are the properties such as surface topography, surface roughness, surface chemistry, surface energy, wettability, thickness of the Ti oxide layer and the presence of impurities [32]. To achieve the aforementioned properties, commonly used surface modifications of the Ti implants are plasma-spraying, acid etching, sandblasting, anodization, inorganic and organic coating, electropolishing, plasma ion implantation, and laser-beam micromachining [33,34].

The last technology mentioned, in particular, shows great potential for the flexible fabrication of miniaturized components and surfaces of specific properties made of various types of materials as is documented in many current studies. The surface functionality by ultrashort pulsed laser texturing has received considerable attention from researchers in the past few decades [35,36,37,38,39,40].

These techniques improve osseointegration—the structural and functional connection between bone and implant—as well as cell attachment, and have an impact on the shortening of healing time [32,34]. Greatly roughed implant surface supports anchorage between cells and implant, which allows ingrowth of the tissue [41]. Many studies [42,43,44,45,46,47,48,49,50,51,52,53,54] have investigated the appropriate surface roughness of implants, however, the ideal and universal roughness value has not yet been found. The suitable surface roughness depends on the cell size; e.g., for the osteoblasts, the adequate roughness Ra is 1–2 µm [53,54]. Roughness values between 1 and 2 µm are also considered to be the necessary roughness for the success of long-term implants and supported prostheses. No specific surface topography has been found to be the ideal surface in allowing the least bacterial biofilm attachment [55].

Several studies have revealed that the research into biocompatible materials and their processing with the goal of optimizing the topographical features of the functional surfaces of implants is still of utmost importance, since many questions concerning the optimal surface morphology have not been answered yet. Combined with growing interest in the use of Ti powder metallurgy (PM) as a cost-effective way of direct production of complex parts made of Ti and its alloys [56], this fact has led the authors to study the laser micromachining of the Ti–graphite composite samples, prepared by pioneering a low temperature powder metallurgy technique. The influence of laser energy on the machined surface morphology, roughness, and chemistry were investigated and evaluated in this study and discussed from the point of view of application in dental implantology.

## 2. Experimental

### 2.1. Experimental Material

**Figure 1:**
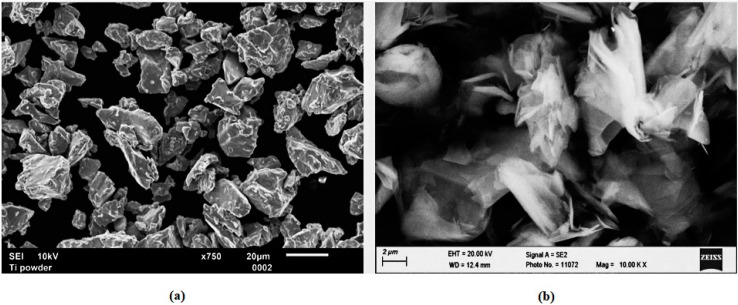
Characteristic shape of used HDH titanium powder and graphite: (**a**) HDH Ti powder particles at the magnification of 750×, (**b**) graphite flakes at the magnification of 10.00k×.

Experimental samples of TiMMC were prepared from the CP HDH (hydrogenated-dehydrogenated) titanium powder (Kimet Special Metal Precision Casting Co., Ltd., Hengshui, China) and 15.0 vol.% of graphite flakes. The Ti powder of particle size below 32 µm exhibits a typical fragmented angular shape which is due to the HDH preparation method (Figure 1a). The graphite flakes of average particle size of 16 µm with aspect ratio of flakes 10 (diameter to thickness ratio) and purity 99.9% are shown in Figure 1b. The powder was compacted using cold isostatic pressing followed by hot vacuum pressing at the temperature of 450 °C and pressure of 500 MPa [57]. The density of samples was determined from weighting and volume measurement to be in the range of 4.1–4.15 g. cm^−3^. Porosity of the finished compact was 2.44% ± 0.15%. The specimen was cut into rollers with a diameter of 24 mm and a height of 5 mm. The rollers were encapsulated by compression mounting into the conductive copper-based ProbeMet mounting compound, and then their surfaces were ground on abrasive grinding papers of the 1200 grit size. The mounted and ground specimens were prepared for laser-beam micromachining.

### 2.2. Experimental Procedure

**Figure 2:**
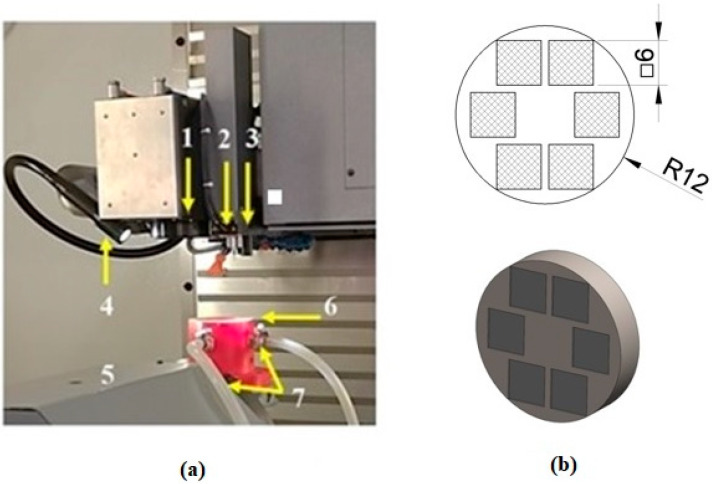
Experimental setup: (**a**): experimental workplace; (**b**): sample with 6 experimental surfaces 1–beam guidance with scanner, 2–Z-level measuring probe, 3–positioning and measuring CCD camera, 4–lighting, 5–kinematics of working table, 6–fixture, 7–shielding gas inlet.

**Figure 3:**
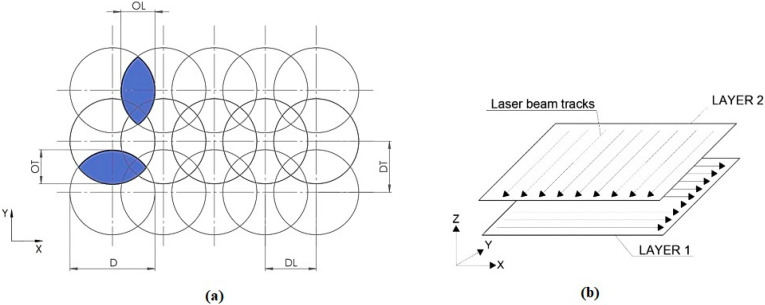
Scheme of the laser beam micromachining motion strategy. (**a**) scheme of pulses overlaps, where D is diameter of laser spots, D_L_ is lateral pulse distance, D_T_ is transverse pulse distance, O_L_ is lateral overlap and O_T_ is transverse overlap; (**b**) scheme of ablated layers.

In this study, the Lasertec 80 Shape machining centre (DMG MORI GmbH, München, Germany) equipped with a nanosecond fibre ytterbium-doped laser operating at 1064 nm wavelength was employed (Figure 2a). Six square-shaped surfaces of 6 mm side length (Figure 2b) were ablated by combining different levels of pulse energy (E_p_) and lateral pulse distance (D_L_) leading to different lateral pulse overlaps (O_L_) (Figure 3, Table 1). This resulted in different amounts of thermal energy density.

During the experiment, a constant pulse duration of 120 ns and a laser spot diameter (D) of 50 μm were set up. A bi-directional traces layout (cross-hatching strategy) was performed with a transverse track distance of pulses (D_T_) of 10 µm, which represents transverse pulse overlap (O_T_) of 80% (Figure 3). The total number of incident laser pulses (N) and the total energy delivered to the material per illuminated area (E_T_) in one layer can be calculated as:

N = D/D_L_ × D/D_T_ (1)

E_T_ = E_p_ × N (2)

The material was ablated in two layers in Ar shielding atmosphere with the gas flow rate of 10 L·min^−1^.

### 2.3. Surface Characterization

Subsequently, the machined surfaces were analysed by scanning electron microscopy (SEM analysis), using the JEOL JSM 7600F (JEOL Ltd., Tokyo, Japan) scanning electron microscope with resolution of 1.5 nm (1 kV) in a gentle beam mode and 1.0 nm in 15 kV; magnification from 25 to 1,000,000 times. The energy-dispersive X-ray spectroscopy (EDS) analyser integrated in the SEM was used for the qualitative and semi-quantitative estimation of chemical composition in three different areas of each machined surface. The area surface roughness parameters were measured on the ZEISS LSM 700 (Carl Zeiss Microscopy GmbH, Jena, Germany) confocal microscope in accordance with the ISO 25,178 Standard. Colour 3D maps of surface were processed in ConfoMap ST software (developed by Digital Surf, Besançon, France). The measured area was 1.18 × 1.18 mm^2^. The measured area roughness parameters were the arithmetical mean of the height of surface (Sa), maximum height of surface (Sz), maximum peak height (Sp), maximum pit height (Sv) and root mean square height of surface (Sq). Finally, the X-ray diffraction (XRD) patterns measurement using a Brucker D8 diffractometer (Brucker, Billerica, MA, US), Cu-Kα X-rays of wavelength λ = 1.5406 Å was used to verify phases transformations. The data was taken for the 2θ range of 15° to 105° with a step of 0.05°.

Statistical evaluation of the oxygen content in the machined surface layers by one-way analysis of variance (one-way ANOVA) followed by Tukey pairwise comparison tests was performed by applying Minitab v. 17 software (Minitab, LLC, State College, PA, US). For the aforementioned statistical tests, the levels of significance were set at 95% (α = 0.05) and 99% (α = 0.01).

## 3. Results

### 3.1. Scanning Electron Microscopy (SEM) Observation Results

**Figure 4:**
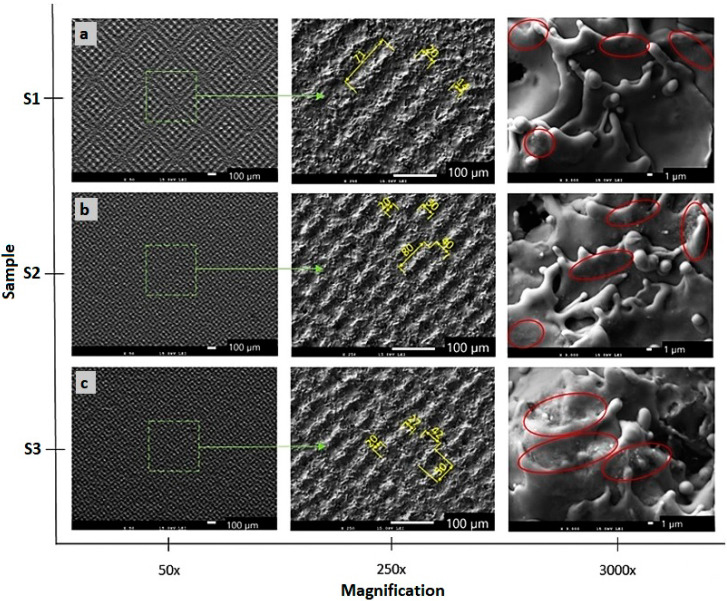
Scanning electron microscopy (SEM) micrographs (original magnifications of 50×, 250× and 3000×) of the machined surfaces. (**a**) surface S1 (total energy delivered to the material per illuminated area (E_T_) = 0.5 mJ); (**b**) surface S2 (E_T_ = 1 mJ); (**c**) surface S3 (E_T_ = 5 mJ). Areas containing oxides are indicated in red.

**Figure 5:**
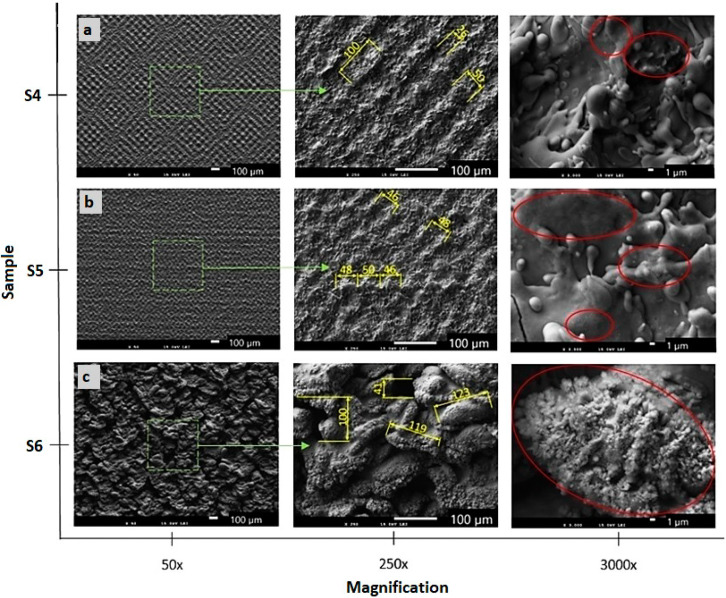
SEM micrographs (original magnifications of 50×, 250× and 3000×) of the machined surfaces. (**a**) surface S4 (E_T_ = 12.5 mJ; (**b**) surface S5 (E_T_ = 25 mJ); (**c**) surface S6 (E_T_ = 500 mJ). Areas containing oxides are indicated in red.

The SEM micrographs of the machined surfaces are presented in Figure 4 and Figure 5. Both pictures capture the surfaces machined, applying different energies at magnifications of 50×, 250× and 3000×.

A melted and rapidly solidified layer of material is visible on all surfaces. Three types of surfaces were observed, depending on the energies used. The first one is a rough surface with porous structure formed by the highest total energy delivered to the material per area (Figure 5c). The second type of structure is formed by the protrusions forming a small square-shaped texture (Figure 4b,c and Figure 5b. The third type of surface is formed by visible laser beam paths that form squares as a result of the cross-hatching strategy (Figure 4a and Figure 5a).

### 3.2. Surface Roughness Measurement Results

**Figure 6:**
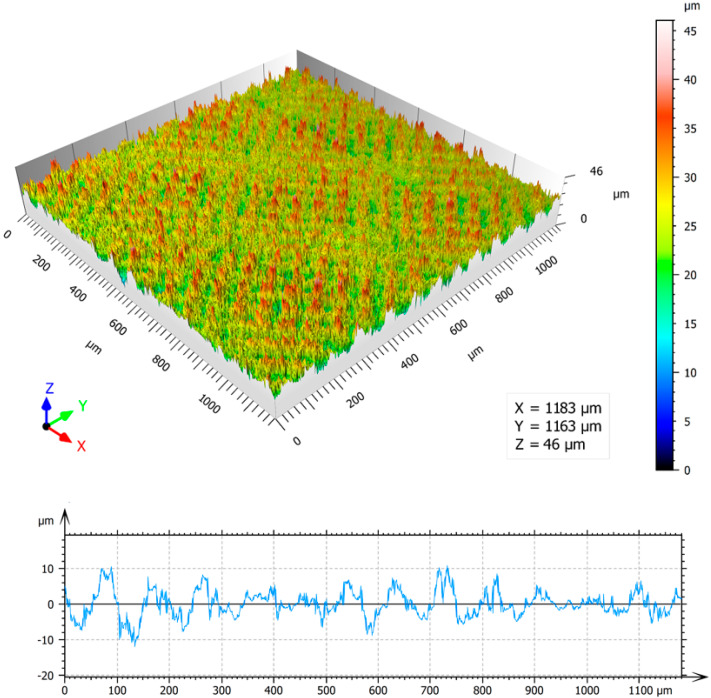
The area roughness measurements–3D map and profile of surface S1: arithmetical mean of the height of surface (Sa) = 3.22 μm, maximum height of surface (Sz) = 46 μm, maximum peak height (Sp) = 22.5 μm, maximum pit height (Sv) = 23.5 μm, root mean square height of surface (Sq) = 4.08 μm.

**Figure 7:**
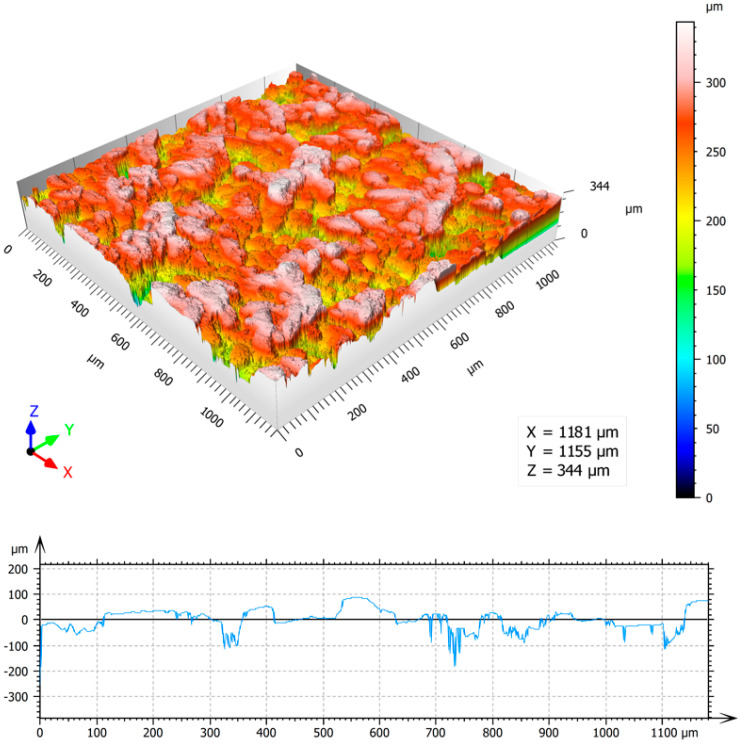
The area roughness measurements–3D map and profile of surface S6: Sa = 33.1 μm, Sz = 344 μm, Sp = 85 μm, Sv = 259 μm, Sq = 41.5 μm.

Colour 3D maps of the machined surfaces S1 and S6 with surface profilograms in the *x*-axis direction and results of area surface roughness parameters Sa, Sz, Sp, Sv and Sq measurement are in Figure 6 and Figure 7.

It is visible that the value of surface roughness parameter Sa of the samples S6, compared with the surface S1, increased approximately by 10 times, while increasing the total laser energy per area by 1000 times. It is also obvious that while the values of maximum peak height Sp increased by 4 times, the increasing of the values of maximum pit height Sv is significantly higher, approximately 11 times. Higher value of energy transferred to the irradiated surface leads to the formation of surface profile with deep valleys and flat peaks.

### 3.3. Chemical Composition Analysis Results

**Figure 8:**
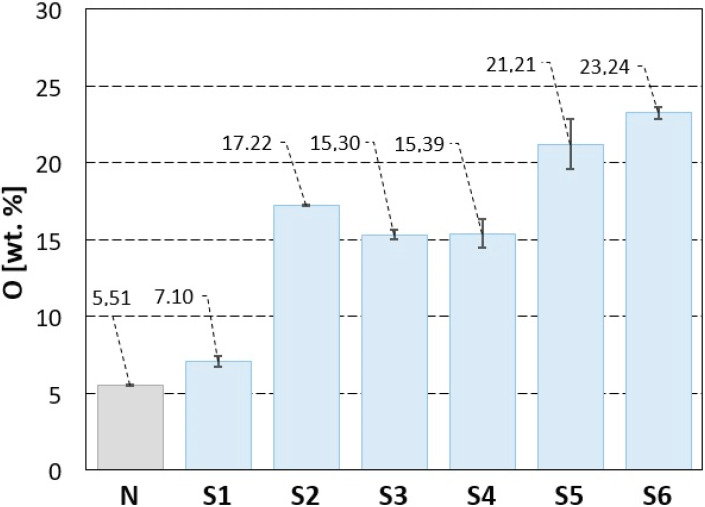
Weight percentage of oxygen content in the non-treated surface (N) and machined surfaces (S1–S6).

Results of the EDS analysis of the machined surfaces are given in Table 2 and Figure 8. They bring means and standard deviations (SD) of titanium (Ti), carbon (C) and oxygen (O), found in all the analysed samples. As can be seen, the minimal average weight percentage of oxygen was detected on non-irradiated (N) surface. The content of oxygen of surfaces S1–S6 increased with increasing value of total energy of irradiation, excluding the surface S2 which was machined using the same value of the pulse energy, but a 5 times smaller value of the total incident energy than surface S3, while the detected content of oxygen on surface S2 is higher than that observed on the surface S3.

**Figure 9:**
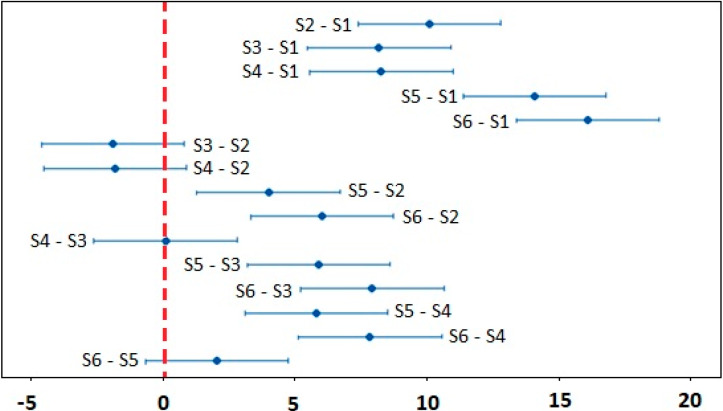
Tukey pairwise comparison test or oxygen content in the machined surface S1–S6.

The results of analysis of statistical significance of the oxygen concentration dependence on the incident energy by one–way ANOVA (Fisher’s test) are documented in Table 3. The alternative hypothesis was confirmed, i.e., at least one mean value of the oxygen concentration exhibits a difference on the significance level of α = 0.05 and α = 0.01. To determine the values which differed significantly, the Tukey pairwise comparisons test was used, the results of which are shown in Figure 9. All confidence intervals not covering zero are statistically different. 

**Figure 10:**
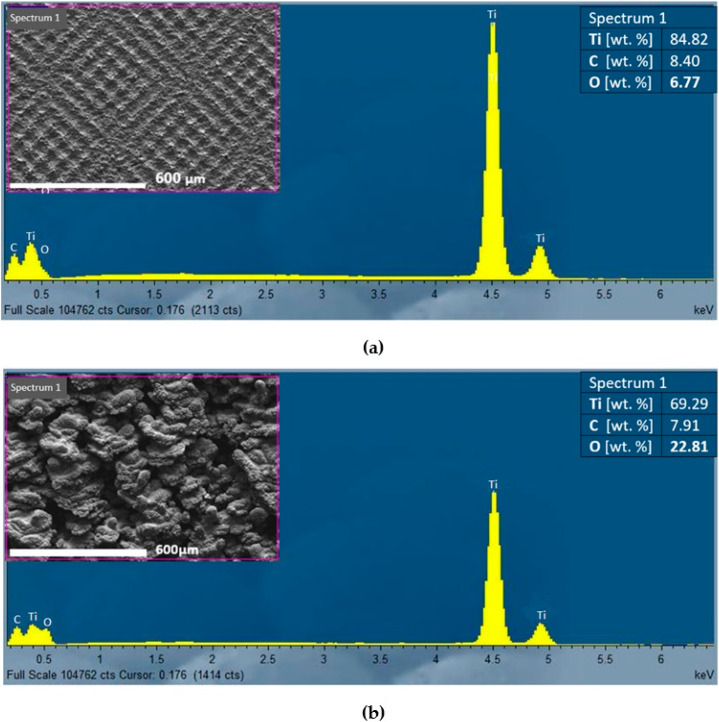
Results of EDS analysis: (**a**) surface S1 (E_T_ = 0.5 mJ, E_p_ = 0.2 mJ, D_L_ = 100 µm), (**b**) surface (E_T_ = 500 mJ, E_p_ = 1 mJ, D_L_ = 0.5 µm).

Figure 10 shows the EDS analysis results for two representative surface types. Figure 10a illustrates the element analysis of the surface S1, machined by pulse energy of 0.2 mJ and lateral pulse distance of 100 μm (minimal total energy E_T_). It is characterized by a texture and a low increase in oxygen on the surface after machining. Figure 10b shows the EDS analysis of the surface S6 that was machined by pulse energy of 1 mJ and lateral pulse distance of 50 μm (maximal total energy E_T_). The surface exhibited a high roughness and a porous structure with the highest oxygen content on the surface.

### 3.4. X-ray Diffraction (XRD) Observation Results

**Figure 11:**
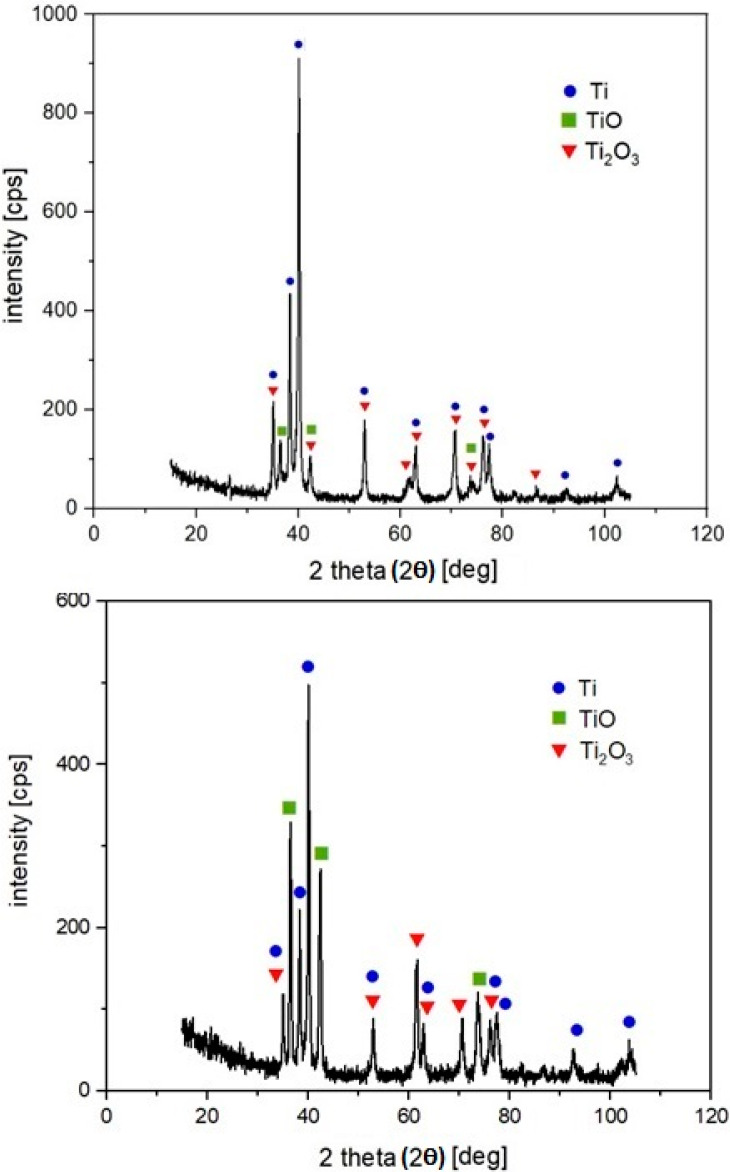
X-ray diffraction patterns of machined surfaces S1 (up); S6 (down).

The X-ray diffraction pattern of the machined surfaces S1 and S6 are depicted in Figure 11. It is evident that the Ti, TiO and Ti_2_O_3_ peaks are visible in the diffractograms. The experimental XRD patterns are well matched with the International Centre for Diffraction Data (ICDD) reference cards n° 03-065-9622 (titanium), n° 01-086-2352 (TiO) and n° 01-071-1047 (Ti_2_O_3_),

## 4. Discussion

Surface morphology of the laser-machined surfaces of Ti-graphite composite prepared by low temperature processing are formed of the ascended ridges of the molten redeposited and solidified globules, craters and cavities. 

At the highest total energy (pulse energy of 1 mJ and pulse overlaps of 99%—surface S6), a rough porous structure containing voids and pockmark was formed. The deep valleys and protrusions of average length of 100 µm were associated with higher ablation intensity, caused by the high value of pulse energy and short lateral pulse distances, when the laser beam strikes repeatedly one place on the machined surface. The greater amount of heat is introduced into the material, the larger volume of material evaporates, causing deep depressions and remnants of molten material to form protrusions. The arithmetical mean height of surface Sa is very high, reaching the value of 33.1 µm. The amount of oxygen measured on the surface reached the maximal value of 23.24 wt.%, as evidenced by the continuous oxide layer in the SEM micrographs (Figure 5c).

The surfaces of samples S3 (E_T_ = 5 mJ) and S5 (E_T_ = 25 mJ) machined by a lateral pulse overlap of 80% (D_L_ = 10 µm) exhibited a similar structure formed by the square-shaped protrusions resulting from the crosshatching strategy. The length of the ascended ridges is 50 µm (Figure 4c and Figure 5b). The oxygen amount in surface S3 was 15.3 wt.%, and in surface S5 was 21.21 wt.%. The islets of oxides were present on the surface. 

Sample S2 shown in Figure 4b and machined by E_T_ = 1 mJ (E_P_ = 0.2 mJ and lateral track distance 50 µm, which represents 0% lateral pulse overlap), exhibited a surface structure of small four-quarter protrusions of a length between 70 and 80 µm, and a width between 40 and 50 µm. The spacing between the protruding parts was 20 µm. The oxides created small discontinuous islets on the machined surface. A regular checked pattern with significant laser beam paths was produced on sample S4 in Figure 5a, machined by E_T_ = 12.5 mJ (pulse energy of 1 mJ and lateral pulse overlap of 60%). The protruding parts showed a length of 100 µm and a width of 50 µm.

A similar gridded square texture as that on surface S4 was also formed on the surface of sample S1, in Figure 4a. The length of the protrusions corresponds approximately to the lateral pulse distance, which represents 100 µm. The width of the protrusions is 20 µm, and they form a regular texture with evenly spaced tapering projections. The highest projection was 22.5 µm, while the arithmetical mean height was the lowest measured value Sa 3.22 µm. The minimum oxygen level of 7.1 wt.% was measured on the surface of sample S1.

The texture visible on the surfaces S1 and S4 copies the laser beam traces. It is documented when the maximal scanning speed of 2000 mm·s^−1^ was used despite the different values of pulse energies and lateral pulse distances.

The process of surface morphology formation was strongly affected by the amount of thermal energy concentrated in the unit volume of material, which is controlled by the laser machining parameters setting, including laser power, pulse frequency and scanning speed. Different combinations of the three parameters give different pulse energies and lateral pulse distances resulted in the different total incident energies per machined surface. The total energy is used as the controlled process parameters in this study. It led to the melting of a different volume of material as well the formation of different heat-affected zones.

Machined by applying higher energies, surface S6 revealed rough surface structures. Several irregularly shaped macro and micro cavities observed on the machined surface were the original porosity of the material, that together with the craters and microscale irregularities, having resulted from the melting and partial melting of the machined material, provided a surface structure beneficial to bone cell attachment, new bone integration and adhesion between the bone tissue and implant, as have been confirmed by many authors [58,59]. By roughening the implant surface, the healing of the bone can be improved significantly. On the other hand, a very important requirement regarding the implant surfaces are their antibacterial properties [60]; therefore the surface structures with the features slightly smaller than the bacteria size, or the hole/crater-like structures slightly larger than the bacteria size, are used. As documented in this study, the moderately rough surfaces can be produced by laser machining when using lower energy densities (surface S1).

The high thermal fusions in machined material during laser machining cause its superficial melting accompanied with the oxygen diffusion through molten material and the subsequent formation of a specific oxidic layer on the surface, which is an example of gaseous corosion of the material occurring at high temperatures. The oxygen content in a machined surface results from residual content of oxygen in the shielding gas and the gas supply manner. The higher values of used energies correspond to higher content of oxygen detected on the machined surface. In addition to titanium peaks, the XRD pattern confirms the presence of two types of oxides, TiO and Ti_2_O_3_. The obvious type of oxide TiO_2_ (rutile or anatase) was not confirmed in any case. Only during the preliminary experiments conducted without shielding Ar gas, did we observe the XRD peak at around 25.3° which could correspond to TiO_2_ (anatase). It can be assumed that, as the most thermodynamically stable form of oxide, TiO_2_ was probably present on the surface in an amorphous structure which was formed due to the very rapid cooling of liquid metal, as was documented also in [61]. It is in agreement with [62], whose authors stated that oxidation products’ composition may be very complex, changes gradually, and is a function of the process parameters.

The results of the study confirm that the PM processed composite materials with improved mechanical properties together with laser surface micromachining offer a cost-effective solution for application in dental implantology.

## 5. Conclusions

In this research, PM-processed titanium-graphite composite was machined by laser beam micromachining in Ar atmosphere. On the basis of this research, the following conclusions can be drawn:

(1) It was found that a regular texture on the PM-processed composite material can be achieved via a suitable combination of the input parameters of the laser micromachining process.
(2) The machined surface is formed of ascended ridges of the molten redeposited and solidified globules, craters and cavities. The documented deep valleys and protrusions were associated with higher ablation intensity.
(3) EDS analysis detected oxygen on all samples. As the pulse distances increased, the amount of oxygen on the surface decreased slightly. The presence of two types of oxides, TiO and Ti_2_O_3,_ was confirmed on all surfaces by XRD analysis. TiO_2_ was not confirmed in any case.
(4) Statistical analysis using the one-way ANOVA and Tukey pairwise comparison tests revealed which surfaces showed a statistically significant difference in the amount of oxygen on the surface.
(5) The lowest achieved arithmetical mean height Sa was 3.22 µm, at the largest lateral pulse distance, represented by visible traces of the laser beam.
(6) The introduction of more heat into the workpiece material at the same location resulted in the formation of deep valleys which were due to evaporation of the material.
(7) This study helps to identify the laser-beam energy parameters to achieve a pre-defined surface geometry. The lower level of total energy (lower pulse energy together with higher lateral pulse distances) is recommended to achieve beneficial antibacterial surface properties. Higher total energies (higher pulse energies and smaller pulse distances) are suitable for the production surfaces which can improve the healing of the bone
(8) However, the contribution brings only partial insights into an otherwise broad problem. For a wider application of the studied material, therefore, it is necessary to carry out a further series of experiments, especially focused on precise description of the relation between surface structure and osseointegration phenomenon.

## Figures and Tables

**Table 1:** Input process parameters of the laser-beam micromachining.

Machined Surface	Pulse Frequency (kHz)	v_s_ (mm·s^−1^)	D_L_ (μm)	E_P_ (mJ)	N (–)	E_T_ (mJ)
S1	20	2000	100	0.2	2.5	**0.5**
S2	20	1000	50	0.2	5	**1**
S3	100	1000	10	0.2	25	**5**
S4	100	2000	20	1	12.5	**12.5**
S5	100	1000	10	1	25	**25**
S6	100	50	0.5	1	500	**500**

**Table 2:** Results of energy-dispersive X-ray spectroscopy (EDS) analysis of non-treated surface (N) and machined surface (S1–S6).

Sample	Repetitions	Ti (wt.%)		C (wt.%)		O (wt.%)	
		Mean	SD	Mean	SD	Mean	SD
N	3	86.35	0.06	8.15	0.07	5.51	0.02
S1	3	84.32	0.56	8.58	0.22	7.10	0.35
S2	3	72.48	0.21	10.30	0.22	17.22	0.02
S3	3	75.18	0.56	9.52	0.28	15.30	0.30
S4	3	76.66	1.30	7.95	0.24	15.39	0.92
S5	3	68.52	1.99	10.26	0.41	21.21	1.64
S6	3	67.29	3.13	10.14	3.70	23.24	0.41

**Table 3:** One-way analysis of variance (ANOVA) results.

Samples	DF	F-Value	*p*-Value	R^2^	Pooled SD
S1–S6	5	97.48	0.000 *	97.60	0.9896

*At least one mean is different for significance level α = 0.05 and α = 0.01.
